# Supplementary material for: petiteFinder: an automated computer vision tool to compute Petite colony frequencies in baker’s yeast
Source: BMC Bioinformatics. 2023 Feb 15;24:50. doi: 10.1186/s12859-023-05168-5 (PMC9930278; doi:10.1186/s12859-023-05168-5)
Supplement: Supplementary file 1 — Additional file 1. Appendix A, B, and C for petiteFinder. [file 12859_2023_5168_MOESM1_ESM.pdf]

Appendix A

Our semi-supervised colony detection approach

A semi-supervised Grande/Petite colony detection approach as a stepping stone towards *petiteFinder*.

A.1 Description of the labeled dataset (smaller and fewer media types than *petiteFinder*)

The dataset used in this project is a collection of 59 petri dish images of yeast colonies on synthetic dropout media with glucose (0.1%) and glycerol (3%) as carbon sources. Mated Grande and Petite colonies were plated onto this media, with Petite progeny showing up as smaller less opaque colonies compared to their Grande counterparts after 3-5 days of incubation at 30°C. Six plates at a time were scanned after the same period of growth on a computer scanner. They were then cropped to images containing an individual plate with the aid of a 3D printed scanner insert that fixed their positions and also reduced refraction due to adjacent plates. These images were annotated using the LabelMe package [1], where bounding boxes were drawn around Grande and Petite colonies and assigned to their corresponding labels. In total 4868 bounding boxes are in this dataset, with 2684 Petite and 2184 Grande labels. Variation in agar concentrations within the media poured into plates in this dataset produced 20 images we consider "non-ideal" due to the diffuseness of colonies, and 39 images we consider "ideal". For the purposes of testing the predictive capacity of an unsupervised object classification pipeline we separated "ideal" and "non-ideal" images into

80% validation sets and 20% test sets. See Figure A.1 for a summary of this dataset.

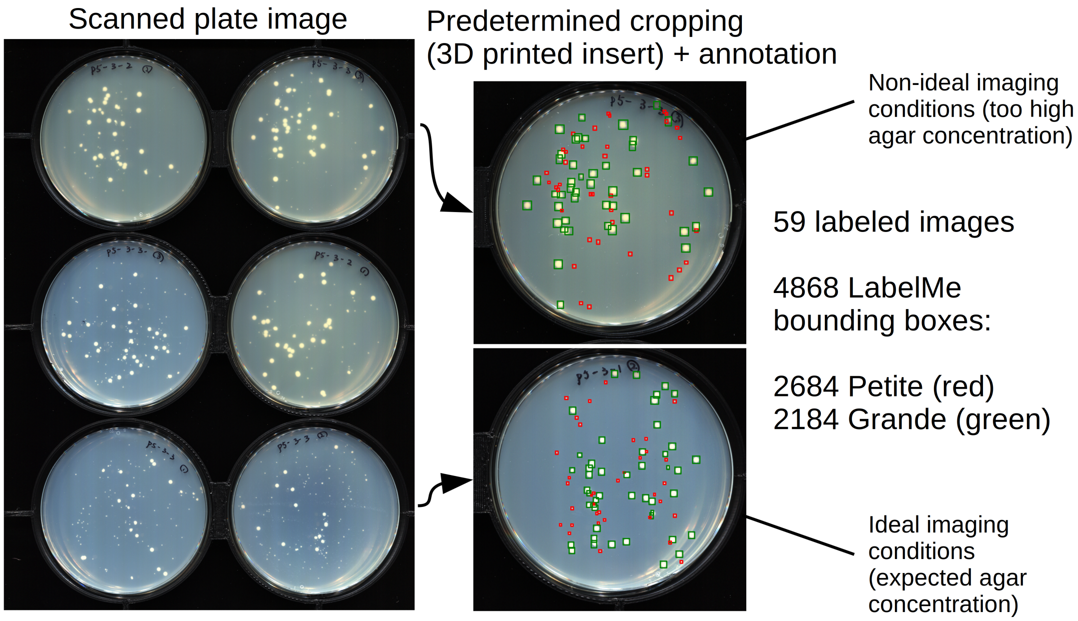

Figure A.1: **An overview of the labeled dataset for the semi-supervised approach.** Petri dishes of synthetic complete media and Grande/Petite yeast colonies were placed into a 3D printed insert and scanned on a computer scanner bottom-up (leftmost image). Individual plate images were cropped out of the large image, and 59 of these images were annotated using the LabelMe annotation tool [1]. Grande and Petite colonies are indicated by green and red bounding boxes, respectively. Experimental variation in media preparation/pouring of plates produces diffuse low quality scans and sharper plate images as shown by the upper and lower plate in the middle panel.

A.2 An unsupervised Petite colony detection approach

A.2.1 Feature selection and clustering of labeled data

We built the core of the unsupervised classification algorithm by selecting distinguishing features of Grande/Petite colonies expected from biological understanding. Petite colonies are smaller than Grande colonies due to the inability of Petite cells to switch to respiration once carbon sources are consumed. This also means that the density of cells in Petite colonies should be lower than Grandes because respiring cells are unable to grow on top of the region of consumed glucose. This suggests that differences in both the size of Grande and Petite colonies and their pixel intensities might enable their classification. To test this idea, we took all 59 annotated images in the labeled dataset and clustered the annotated bounding boxes surrounding Grandes and Petites in size and intensity (Figure A.2). Figure A.2a shows an example plate image with bounding boxes around colonies. Figure A.2b shows the average-linkage hierarchical clustering with Euclidean distance of

these bounding boxes in size and intensity, where clusters with a larger average size are considered Grandes. This clustering method was selected because there was no implicit assumption of equal cluster sizes, and it was sufficiently sensitive to outliers which is important for plate images with low/high Petite frequencies.

We evaluate the effectiveness of this classification scheme on all 59 plate images by considering the precision and recall of Petite classification (Figure A.2c). The precision is $\frac{TP}{TP+FP}$, where TP are the true positive classification counts and FP are the false positive counts. The recall is $\frac{TP}{TP+FN}$, where FN stands for the false negative counts. The precision and recall for Petites across all 59 images is 0.933 and 0.999, respectively. The Grande precision and recall is 0.999 and 0.912, respectively. These metrics suggest that the classification scheme struggles with false positives Petite classifications, which are due to Grande colonies being misclassified as Petite colonies. In Figure A.2d we show the Petite frequencies predicted per plate according to this scheme compared to the ground truth from annotated data. It highlights that while we have good average Petite classification accuracy (average deviation of 0.038 in Petite frequency, standard deviation of 0.058), plates where Grande colonies are overwhelmingly classified as Petite can produce significant deviations from ground truth measurements. Overall, however, these results were promising and suggested that this type of classification scheme combined with a colony detection pipeline may perform reasonably well. We discuss the construction of this joint colony detection and classification pipeline in the next section.

A.2.2 Combining colony detection and classification

In order to create an automated computer vision pipeline that accepts plate images and computes Petite frequencies we needed to combine methods to detect colonies and the previously explored classification scheme. To detect colonies, we opted to use a thresholding approach to separate colonies which are brighter in images from their darker uniform background on the agar surface (Figure A.3). To start, we apply a Hough transform [2] to identify the plate edge with an estimated plate radii range provided by the user. An example plate edge detection is shown in red in the upper right panel of Figure A.3. This allows us to mask out the exterior of the plate and restrict the colony detection to real ones on the agar surface. This is followed by Otsu's thresholding [3], which is an algorithm that selects a foreground/background intensity threshold by finding the value that minimizes the intensity variation within each class. Given maximum ($args.max$) and minimum ($args.min$) expected colony sizes provided by the user, we then apply a watershed transform [4] with a minimum separation between minima of watershed basins of $\sqrt{\frac{args.max}{4\pi}}$ to focus on separating

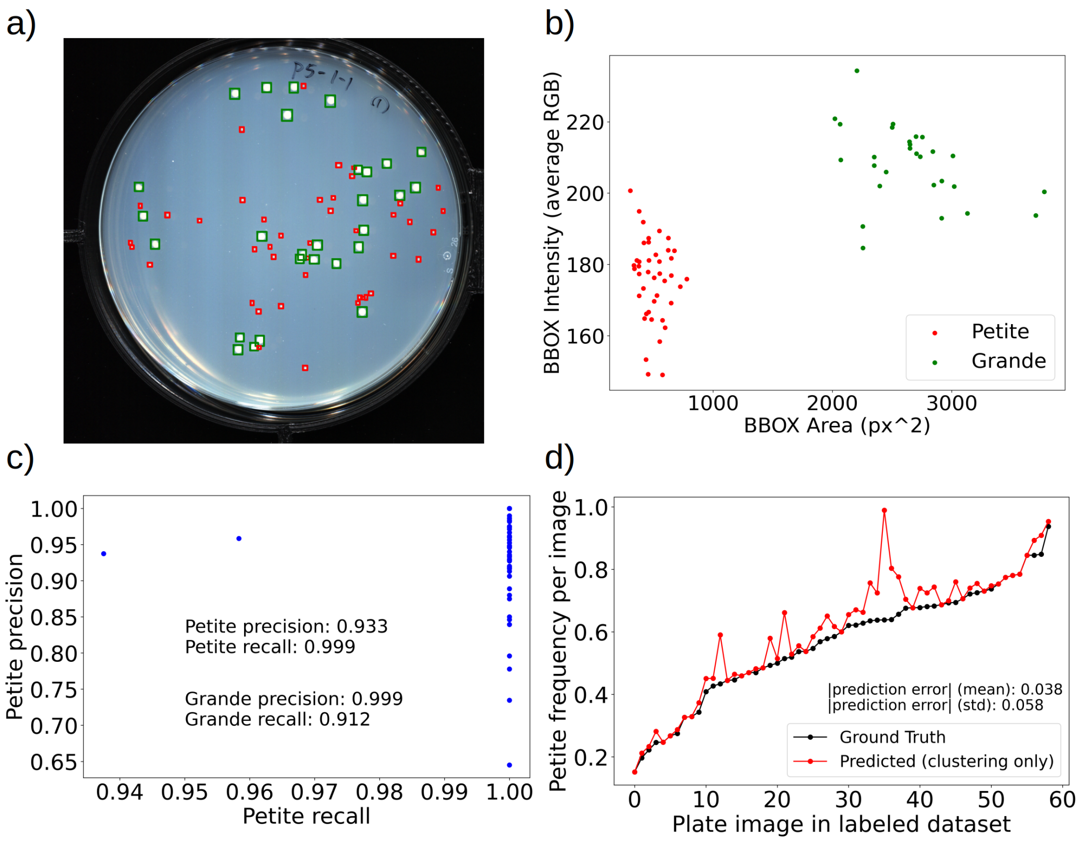

Figure A.2: **Performance of unsupervised average-linkage hierarchical clustering on the bounding boxes of labeled data.** **a)** An example plate image with annotated bounding boxes around colonies generated through the LabelMe annotation tool. **b)** Average-linkage clustering result of bounding boxes (BBOX) in area and average intensity of the contained pixels from the image in (a). **c)** Precision and recall of this unsupervised classification of Petite bounding boxes across all 59 labeled images. Each dot represents the precision and recall of each plate image for Petite classification. Also noted are the precision and recall for Grandes which are not shown. **d)** The Petite frequencies computed per plate in the labeled data using this clustering approach as well as the ground truth petite frequency determined through human annotation. Average and standard deviation in prediction error is also denoted, where prediction error is the difference between ground truth and predicted petite frequencies per image.

merged Grande colonies. This is followed by the removal of colonies with sizes above $args.max$, below $args.min$, and with eccentricity > 0.9. Filtering out only high eccentricity objects we found removes fabric fibres from images, but retains segmented merged colonies that don't necessarily have an eccentricity near zero. Finally, segmented regions are clustered according to the previous section assuming that they are colonies.

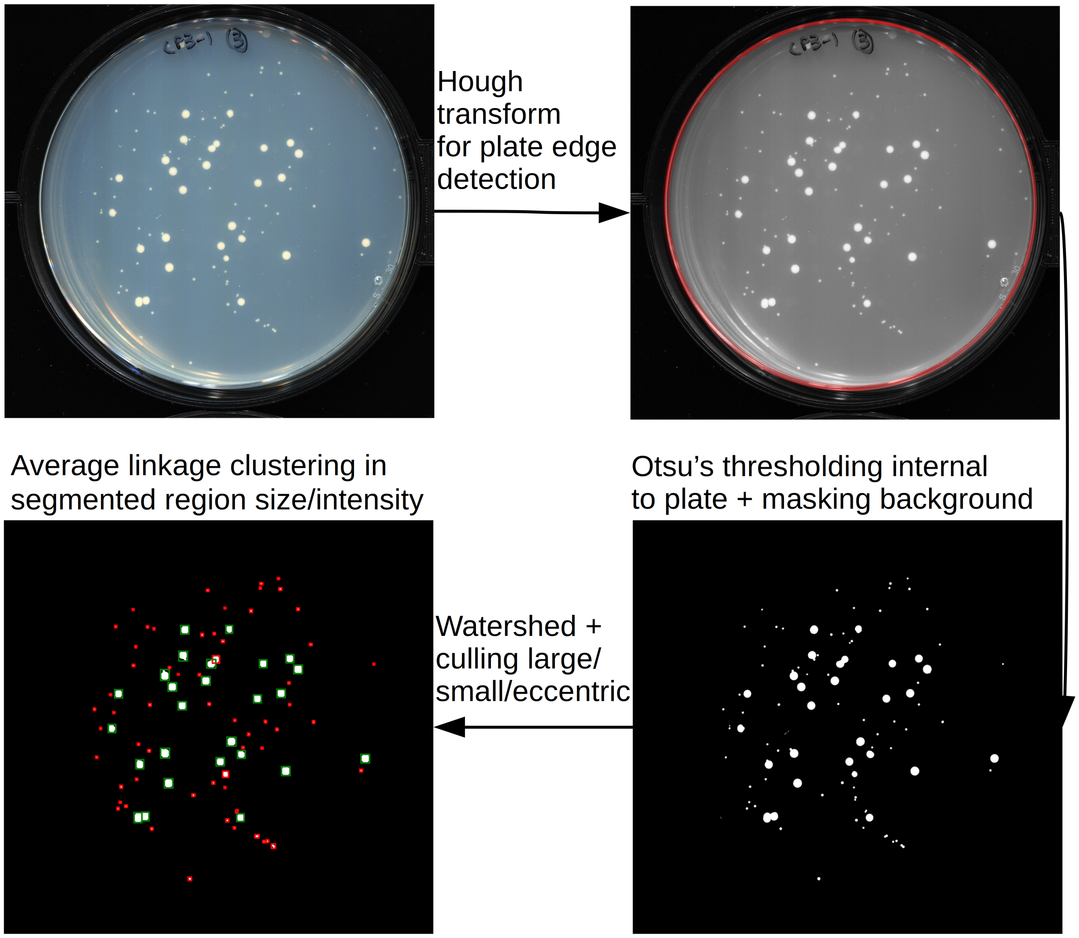

Figure A.3: **An overview of an unsupervised automated colony classification pipeline that identifies colonies and classifies them as Petite or Grande based on the clustering approach detailed in Figure A.2.** A Hough transform accepts a user suggested radius parameter (to account for various plate and image sizes) and outputs the predicted plate boundary in red. A mask is applied to the exterior of the plate, followed by Otsu's thresholding to segment the image into foreground (white), and background (black). Given a maximum ($args.max$) and minimum ($args.min$) expected colony size provided by the user, a watershed transform is applied to the foreground with a minimum displacement between basins of $\sqrt{\frac{args.max}{4\pi}}$ to separate only the merging of large colonies which is most common. Colonies with size above $args.max$, below $args.min$, and with eccentricity > 0.9 are removed. Finally, the labeled foreground objects are clustered using average-linkage clustering in size and intensity features of the labeled region.

A.2.3 Evaluation of the combined colony detection and classification pipeline

To evaluate how effective this complete Petite classification pipeline is we must first address three important points:

The first is that $args.min$ and $args.max$, the expected minimum and maximum colony sizes, must be selected for the set of data we are testing this pipeline on. We do this by testing numerous ranges of these parameters for the "non-ideal" and "ideal" validation image sets and picking the

parameter values that maximize performance by computing the F_1 score $(\frac{2 \times precision \times recall}{precision + recall})$. The maximum value of this score indicates the best result across all parameter regimes if precision and recall are equally weighted. With this approach the pipeline becomes semi-supervised, which is similar to users inputting these maximum and minimum colony values per image or batch of images and is common in modern colony detection approaches [5, 6].

The second point is that to compare predicted bounding boxes (the rectangles that bound unique segmented regions from the pipeline), and ground truth bounding boxes from human labeling, we have to consider the overlap of these rectangles. The most common approach is to compute the intersection area over union (IOU) of the predicted and ground truth bounding boxes and provide an IOU threshold above which we consider a good prediction. As will be discussed later, the thresholding approach we took in the pipeline often reduces the size of Petite colonies due to their transparency and therefore similarity to the background. Thus, we choose an IOU threshold of 0.1 above which we consider bounding boxes to overlap to account for the shrinking of Petite colonies after thresholding.

The third point is that to really evaluate the accuracy of this now semi-supervised approach we individually tune colony size parameters in the validation image sets, which is similar to users selecting maximum/minimum sizes per image, but test the predictive value of the pipeline on separate test images.

As a demonstration of the colony size parameter tuning, we show the precision and recall for both Grande and Petite colonies in the "ideal" validation image set (31 images) in Figure A.4 across a variety of minimum/maximum colony sizes. In the computation of recall and precision in this figure we use an IOU threshold of 0.1. In Figure A.4a and Figure A.4b we can see that the optimal maximum and minimum colony area values of 4000 px and 20 px provide an average precision and recall for the detection of Grandes of 0.98 and 0.73 respectively. In Figure A.4c and Figure A.4d we see that the best average precision and recall for Petites are 0.73 and 0.8, respectively.

Given a prescription for how to choose minimum/maximum colony sizes to optimize prediction we now test how predictive the semi-supervised pipeline is for the "ideal" test image dataset (8 images) in Figure A.5. In Figure A.5a we show the precision and recall for Petite and Grande classification as a function of IOU. We can see clearly that Petite colony classifications are more sensitive to IOU thresholds because of the similarity of their intensities to the background intensity. We see reasonably high precision and recall for Petites (~0.8) at an IOU of 0.1, but a Grande recall near 0.6. This is due to either the elimination of Grandes according to the maximum colony size selected or Grandes being classified as Petites. In Figure A.5b we also show the petite frequency predictions

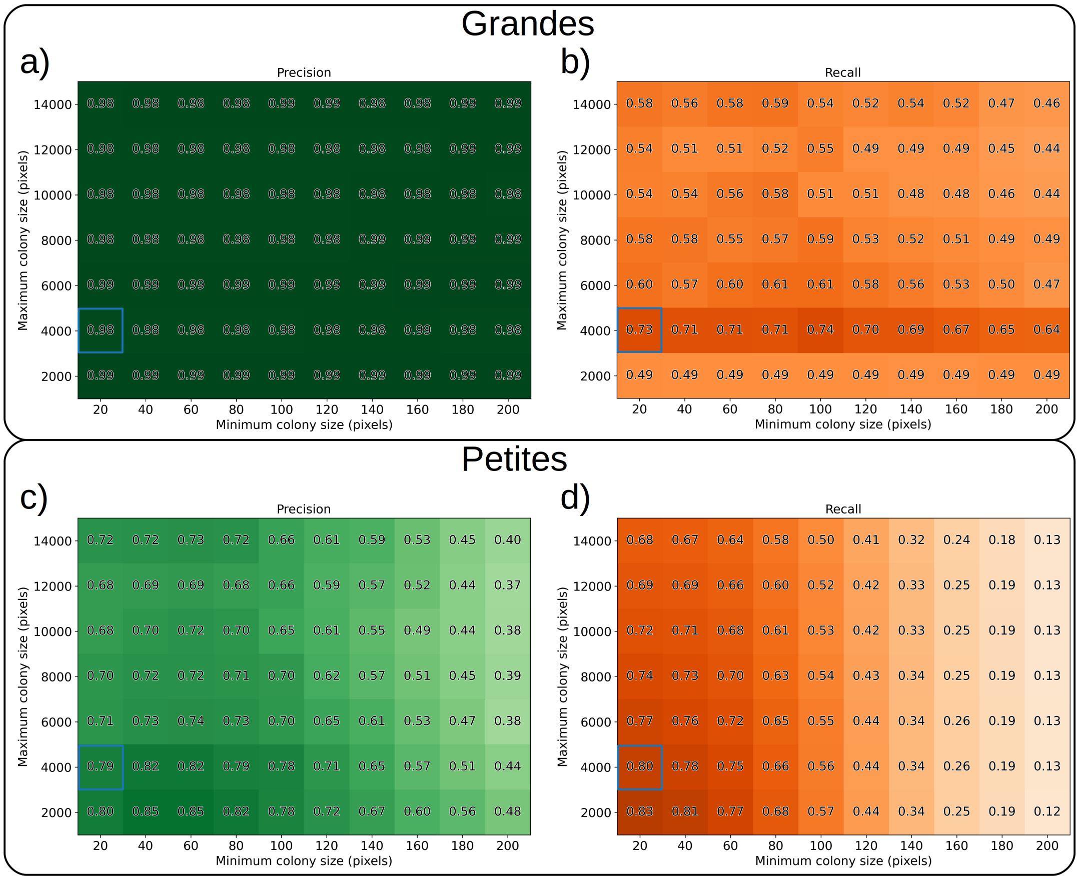

Figure A.4: **Precision and recall of the unsupervised pipeline for both categories across numerous** $args.min$ **and** $args.max$ **parameters for the "ideal" images in the labeled validation set.** The parameter regime that maximizes the F1 score averaged across each category is highlighted in blue. **a)** Precision for Grande colonies. **b)** Recall for Grande colonies. **c)** Precision for Petite colonies. **d)** Recall for Petite colonies.

per image, which indicate that in all but one image we are overestimating Petite frequency. This suggests that we are either classifying dust as Petites, Grandes as Petites, or selectively removing Grandes due to the maximum colony size parameter.

We summarize the effectiveness of this colony detection pipeline in Table A.1, where we include optimal maximum/minimum colony sizes inferred from the "ideal" and "non-ideal" image validation sets and precision and recall in the corresponding test sets. As can be seen from this table, the optimal colony size thresholds vary significantly due to experimental variation and so does the classification accuracy of this approach. This suggests that while we see reasonable Petite/Grande classification especially in the "ideal" test dataset using our approach, parameters like minimum and maximum sizes really do need to be tuned per image or batch of images to obtain the best performance if we are relying heavily on size as a feature. This can be remedied by an automated

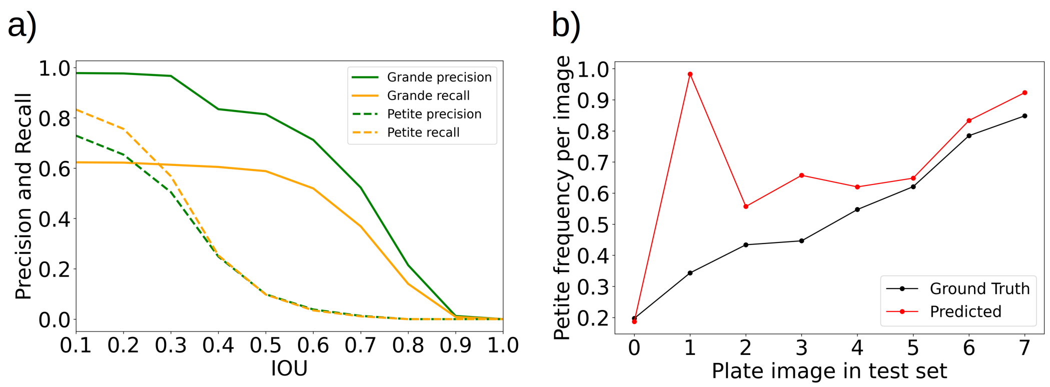

Figure A.5: **Colony detection performance of the semi-supervised pipeline. a)** Precision and recall of both Grande and Petite detection as a function of the intersection over union (IOU) of the predicted and ground truth bounding boxes in the "ideal" test dataset. Here the min/max colony size parameters are those highlighted by the blue boxes in Figure A.4. **b)** Petite frequency predicted by the automated unsupervised pipeline compared to the ground truth human annotations (test set).

deep learning object detection approach, which for example can easily learn other defining features of colonies vs. noise or dust such as nonlinear radial intensity profiles and irregular colony boundaries.

**Image test set (IOU=0.1)**	**Grande precision**	**Grande recall**	**Petite precision**	**Petite recall**	**optimal minimum colony size from validation set (pixels)**	**optimal maximum colony size from validation set (pixels)**
"Ideal" (8 plates)	0.98	0.68	0.75	0.87	20	4000
"Non-ideal" (4 plates)	1.0	0.69	0.66	0.21	60	8000

Table A.1: Precision/recall for both Grandes and Petites in the "ideal" and "non-ideal" test datasets.

Appendix B

Failure modes of semi-supervised colony detection methods

B.1 Low complexity colony detection models require user input for robust performance

In this section we summarize the results in Appendix A, which highlight the dependence of a low complexity semi-supervised model on user input to make good predictions. The performance of classical segmentation approaches (intensity thresholding, edge detection, watershed, to name a few), are highly dependent on experimental conditions. Furthermore, following segmentation, distinguishing colonies from dust or artifacts requires a characterization of their size, shape, colour, and texture. Unfortunately, all of these properties can change based on the imaging setup (resolution, imaging device, orientation), the types of colonies being grown, and other experimental conditions like media type. As we showed in Figure A.5, performing a low complexity model parameter sweep to find optimal parameters on a validation set, then applying this to a test set, results in insufficient performance for our purposes on a diverse experimental dataset. This insufficient performance is a result of sub-optimal model complexity, and is generally remedied by having users visually tune parameters per image, as is the case for OpenCFU [7], AutoCellSeg [5], and CellProfiler [8]. For example, user input is required for plate edge detection in the case of CellProfiler (or a hand drawn mask), and user input on example colony locations in AutoCellSeg. All of these methods also require a maximum and minimum colony size and thresholds on other properties like eccentricity to eliminate

outliers. Furthermore, given segmentations from these types of approaches, users then have to apply their own classification methods which are also accompanied by more tunable parameters.

To address this issue, in the main-text we built a model with sufficient complexity to learn the relationships between colonies and background that captures a wide range of experimental conditions and doesn't require user input to perform well.

B.2 Failures of segmentation and classification

In this section we provide example images to highlight common scenarios where semi-supervised colony detection approaches fail.

We start with example segmentation results from OpenCFU on the *petiteFinder* image test set with the model parameter regimes described in the main-text. Examples of common false negative and false positive segmentation are shown in Figure B.1. False negatives, which are often enriched for Petite colonies, are caused by too high intensity or size thresholds in segmentation (Figure B.1a). Because Petite colonies are semi-transparent, they often are classified as background due to their size and limited contrast with the background. Choosing appropriate colony size and intensity thresholds are especially difficult when the distributions of both of these measures have high variance like they do in the Grande/Petite problem; it is much easier with uniform colony morphology. Figure B.1b shows examples of false positive segmentation. This most often occurs in written text on the surfaces of Petri dishes, in refraction artifacts like the bright arcs in the lower right of the image, or near droplets of condensation. Filtering colonies by shape through measures like eccentricity can remove these types of false positives, although care needs to be taken to not eliminate merged or oblong colonies.

CellProfiler with the recommended model parameters (described in the main text) often results in a high number of false positives, which occur in the same types of areas described above, such as around written texts and refraction artifacts (Figure B.2). CellProfiler also misdetects engraved symbols on the Petri dish as yeast colonies (Figure B.2). Therefore, to manually eliminate many of these false positives, an eccentricity filter is required to improve the performance of the segmentation algorithm.

Next, we show examples where classification fails in our semi-supervised approach tailored from the ground up for the Grande/Petite colony detection problem (described in Appendix A). Figure B.3a shows false negatives, which like OpenCFU are enriched in Petite colonies for the same reasons described above. Figure B.3b shows false positive Petite colonies from two different sources. The

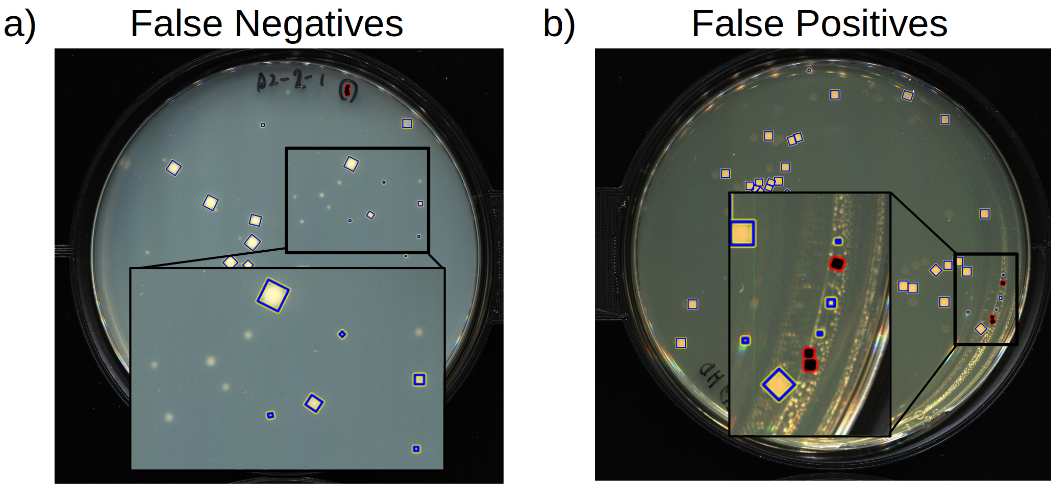

Figure B.1: **Common errors in colony segmentation (OpenCFU).** Images shown here were processed with OpenCFU, with bounding boxes displayed as blue rectangles. Red boxes are those that are removed through outlier detection. Insets show enlarged portions of the images. **a)** Examples of false negative segmentation. While both Grande and Petite false negatives are visible, clearly defined patches of Petite colonies are not segmented from the background. False negatives like these are often enriched in Petites because of their semi-transparency. **b)** Examples of false positive colony segmentation in water droplets and refraction artifacts. Depending on size thresholds, these types of false positives can often be eliminated through filtering colonies by eccentricity.

majority of false positives in the inset (red) are from segmentation of small droplets resulting from condensation inside the Petri dish. These types of artifacts are especially difficult to handle, as droplets are circular and of a similar size to Petite colonies. Center left of the inset of Figure B.3b shows a false positive Petite that results from a refraction arc in the Petri dish.

What do these types of errors mean for the accuracy of computed Petite frequencies? To explore this, we plotted the predicted Petite frequencies across the test image set for OpenCFU plus our unsupervised clustering in size/intensity. The results are shown in B.4 as a comparison between predicted and ground truth frequencies, mirroring Figure 4a from the maintext. Evidently, the best performing semi-supervised approach we tested yields multiple predictions far outside the theoretical sampling error envelope. Figure B.5a (corresponding to image label 15) is an example of a an image where the absolute difference in Petite percentage between ground truth and predicted is 29%. We show the improved results from *petiteFinder* on this image in Figure B.5b. The poor segmentation and classification performance of semi-supervised methods, in addition to the extensive user input required to achieve these results, are what spurred the development of *petiteFinder*.

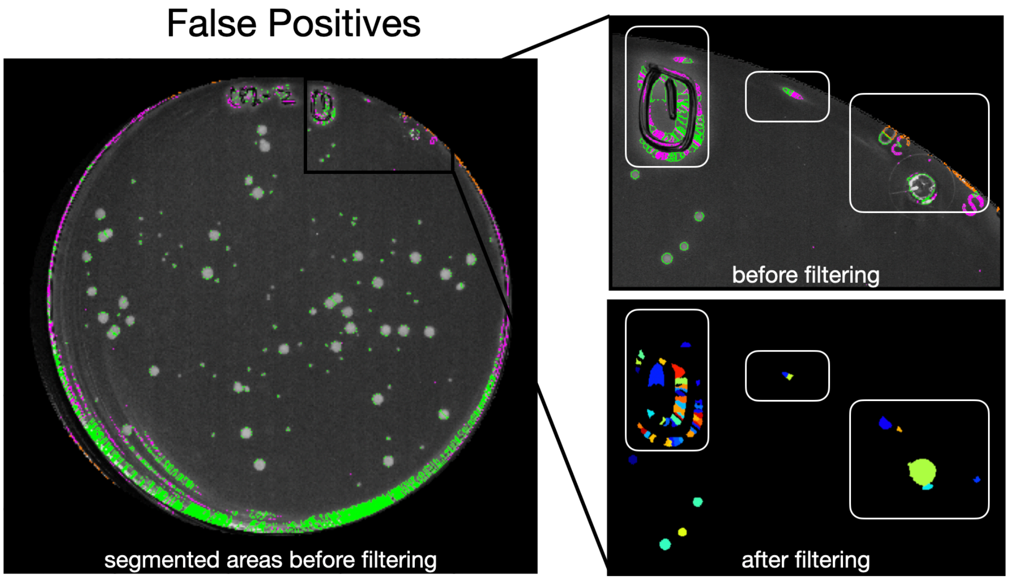

Figure B.2: **Common errors in colony segmentation (CellProfiler).** The images from the test set were processed and visualized with CellProfiler, with colony outlines shown in colors. Insets show enlarged portions of the image before and after colony area filtering. Area filtering helped to remove a lot of small artifacts. Examples of false positive segmentation include areas around written text, refraction artifacts, and engraved symbols on the surface. Some of these false positives can be additionally eliminated through filtering by eccentricity (e.g. < 0.6) at the next pipeline step.

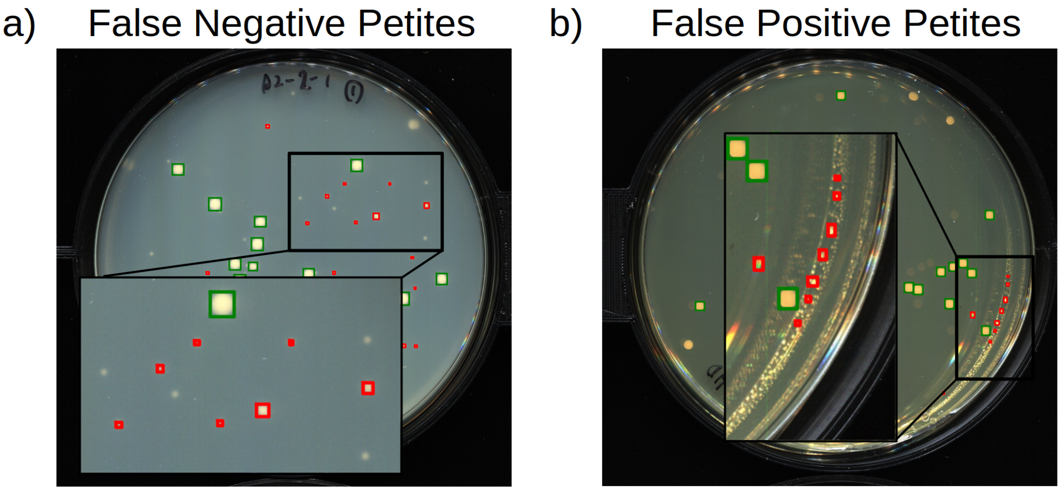

Figure B.3: **Common errors in colony classification (Our semi-supervised pipeline).** Images shown here are processed with the semi-supervised pipeline as described in Appendix A. Insets show enlarged portions of the images where Grande colonies are surrounded by green bounding boxes, and Petites by red bounding boxes. **a)** Examples of false negative segmentation of Petites, which like OpenCFU are more abundant than for Grande colonies. **b)** Examples of false positive Petite colonies. Droplets from condensation are erroneously classified as Petite colonies, and pass size/eccentricity thresholding. Center left of the inset also shows a portion of a refraction arc that is also classified as a Petite colony.

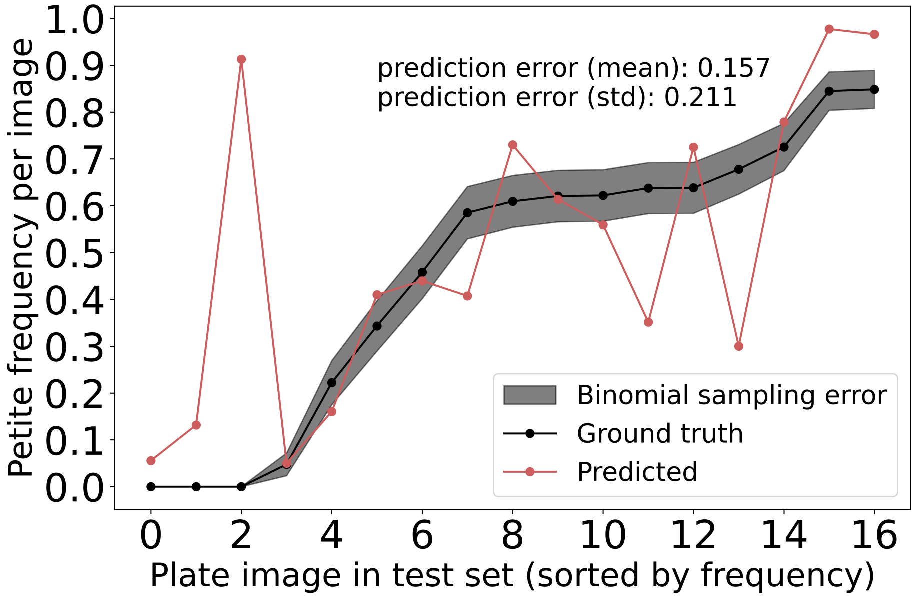

Figure B.4: **Petite frequency predictions from OpenCFU + unsupervised clustering.** A plate-wise comparison of predicted and manually counted Petite colony frequencies from OpenCFU. The red curves are the predictions and the black curves are manual counting. The gray envelope is the binomial sampling error (ground truth ± standard deviation), assuming that Petite production is a Bernoulli process with a probability equal to the ground truth frequency when sampling 78 colonies per plate image.

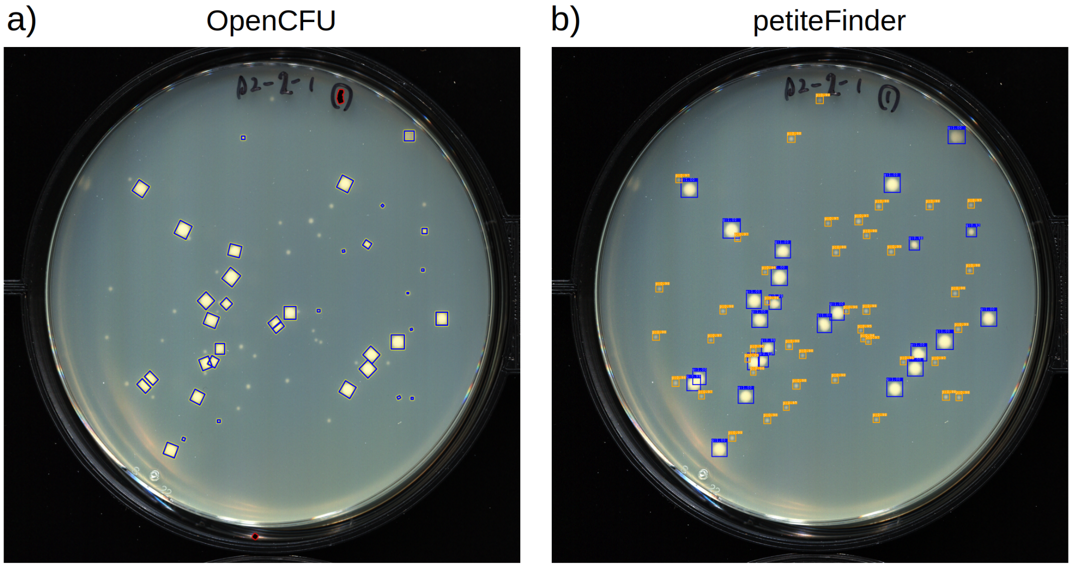

Figure B.5: **Example colony segmentations between OpenCFU and *petiteFinder*. a)** Colony segmentation from OpenCFU on the same image shown in Figure B.1a. Bounding boxes are shown in blue. **b)** Segmentation and classification from *petiteFinder* for the same image, with blue bounding boxes indicating Grande colonies, and orange indicating Petite colonies.

Appendix C

Failure modes of *petiteFinder*

Similar to the semi-supervised methods explored, *petiteFinder* exhibits false positive and negative colony detections from the same types of sources: droplets of water from condensation, refraction artifacts, and writing on Petri dishes. However, as shown by the admirable precision and recall scores across both classes, this occurs significantly less frequently in *petiteFinder* compared to the semi-supervised methods.

What is more interesting is a completely different failure mode of *petiteFinder*, unrelated to imaging artifacts. Part of what the CNN underpinning *petiteFinder* learns during training are the size distributions of Grande and Petite colonies within image slices. To address concerns around this sensitivity to size, which may reduce model robustness, we made sure to have diverse colony sizes within the training and test set across both classes of Grande and Petites. Images were taken after 3-5 days of colony growth, with an equal distribution of growth times, capturing the regular variation in colony size expected during real experiments with yeast. Furthermore, random crops of image slices with a relative size of 0.8 to 1.0 were uniformly applied during training - this transformation enables a wider distribution of Grandes and Petites to be detected. While this experimental variation does improve model robustness, best performance can be expected by adhering to our experimental protocols.

Nevertheless, experimentalists may want to use *petiteFinder* to detect colonies when their size distributions differ as a result of media and/or growth times. How does *petiteFinder* perform under these drastically different conditions? By testing *petiteFinder* on an alternative dataset with large deviations in colony size from the training data, it became clear that the sizes of colonies relative to the image slices are learned and act as distinguishing features between Grandes and Petites.

Furthermore, this realization can be leveraged to improve performance.

In this exploration we sourced an alternative dataset by accessing publicly available images from DOI: 10.5281/zenodo.3779863, the raw images used in DOI: 10.1242/bio.052936. These images of yeast colonies on Petri dishes differ from ours in four important ways:

1. Different yeast strain - The yeast in this alternative dataset are *Schizosaccharomyces pombe*, which grow 2 times more slowly than the *S. cerevisiae* we used. They also are Petite negative, meaning that Petite colonies do not survive. However, *S. pombe* cells with aneuploidy often exhibit smaller, more irregular colonies among wild-type, which can serve as a 'Small' colony class, although they won't have the same size distributions or intensity characteristics as true Petites. Wild-type colonies we will call 'Large'.

2. Different growth times - Yeast colonies in this dataset were grown for 5-7 days at 30°C, which impacts media opacity as well as colony size.

3. Different media - Yeast were grown in YES media, which will have different opacity and colour from the media we used in experiments.

4. Different imaging techniques - Colonies were imaged top-down, using a camera instead of a flatbed scanner, with a lighter background.

Overall, these differing experimental conditions yielded smaller colonies (all colonies were 50% smaller than our Grande colonies relative to image size). There was also no obvious intensity difference observed between the 'Small' and 'Large' colonies, unlike the Petite and Grande colonies in our assay. The latter effect is possibly a result of similar cell density between 'Large' and 'Small' colonies, but also may be related to light being reflected off of these colonies and not transmitted through them as it is during image scanning.

In Figure C.1a we show the default output of *petiteFinder* on an image from this alternative dataset. Segmentation accuracy is remarkably good, as it is in our test dataset. A clear issue, however, is that most colonies are classified as being 'Small' (orange), and rarely 'Large' (blue), whereas to our untrained eyes the 'Small' colonies are less frequent than what is shown here. This isn't particularly surprising, as the relative size of the largest colonies are ~50% smaller than the Grande colonies in our dataset that were used to train the model. Furthermore, we have no expectation that in this media the size distributions of 'Small' colonies are as widely separated as Petite and Grande colonies in our media. The translucency of colonies also do not differ substantially, which the model likely uses in its discrimination between Grande and Petite colonies on our media.

Given this evidence of Petite colony bias (which would become Grande bias in the case of larger colonies), we added a user input parameter to *petiteFinder* to improve this perceived poor classification accuracy. In the case of widely different colony sizes from the training dataset, an assumption in the existing image slice computation is violated; we previously assumed that when resolution changes and physical colony size does not, image slices can be computed to maintain the colony area to background ratio from the training data. This is not the case when colony size extends beyond the size distribution of the augmented training data. To address this, users can provide an optional --grande_size parameter during inference that accepts the approximate diameter of a Grande colony in pixels. This allows us to compute a slice size that maintains the relative size of the colonies in the training data. We can do so by computing image slices as $S_W = S_H = \frac{GD_I}{GD_t} \times 512$. In this equation, GD_I represents the typical diameter of a Grande or 'Large' colony provided by a user, GD_t the typical Grande diameter from the training data (=~50 pixels in our data), and 512 is the size in pixels of the training image slice width or height. This equation serves to enforce the same Grande size to slice size ratio as the training data.

With this small change, the model output on the same image from the alternative dataset is shown in C.1b. While we aren't confident in the characteristics that delineate 'Large' and 'Small' colonies in this dataset (and the dataset is not annotated), this change has shifted the classifications towards predominantly 'Large' as we would expect from the image. The result is a binary classification where the variances of size distributions are smaller within each colony class than the previous image, which would be expected to improve true Grande vs Petite classification under conditions where their sizes vary significantly from the training data (Figure C.2).

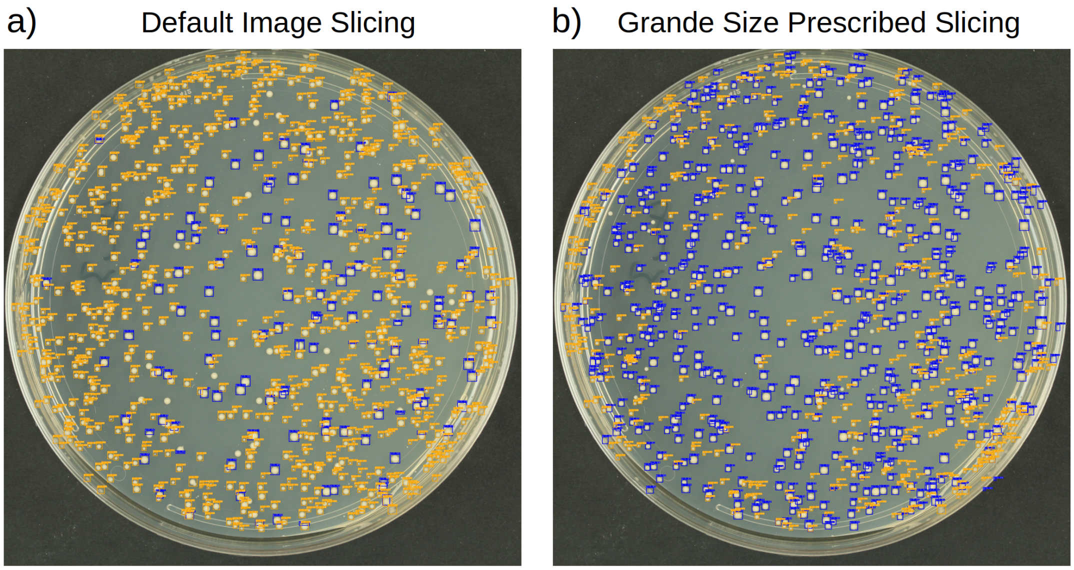

Figure C.1: **Default and user augmented *petiteFinder* output when applied to an image from an alternative dataset.** 'Large' colonies are indicated by blue bounding boxes, 'Small' colonies by orange bounding boxes. **a)** Default output from *petiteFinder* without any user input on a single image from an alternative dataset where the relative size of colonies are 50% smaller than the training data. **b)** The output of *petiteFinder* on the same image when a user defined Grande colony size of 56 pixels is provided.

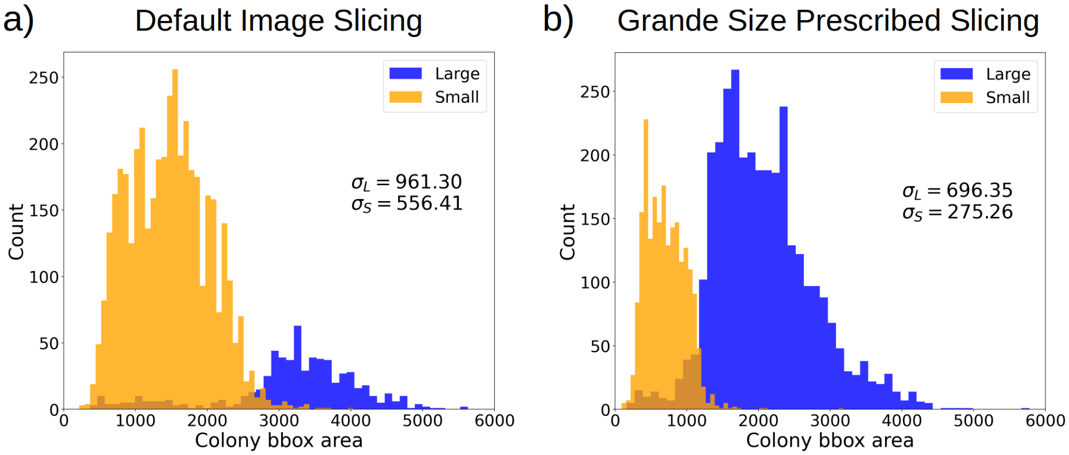

Figure C.2: **Size distributions of 'Large' and 'Small' colonies before and after a user provides a --grande_size parameter in an alternative dataset.** Blue distributions represent 'Large' colony sizes, orange distributions represent 'Small' colony sizes. Distributions are built from colonies across 5 images in the dataset. Standard deviations of the size of each colony class are indicated in the image by σ. **a)** Default colony size distributions from *petiteFinder* without any user input. **b)** Colony Size distributions when a --grande_size parameter with a value of 56 is provided.

References

1. Russell, B. C., Torralba, A., Murphy, K. P. & Freeman, W. T. LabelMe: A database and web-based tool for image annotation. *International Journal of Computer Vision* **77.** doi:10.1007/s11263-007-0090-8 (1-3 2008).

2. Hough, P. V. C. A method and means for recognition complex patterns; US Patent: US3069654A. *US Patent* (1962).

3. Otsu, N. THRESHOLD SELECTION METHOD FROM GRAY-LEVEL HISTOGRAMS. *IEEE Trans Syst Man Cybern* **SMC-9.** doi:10.1109/tsmc.1979.4310076 (1 1979).

4. Digabel, H. & Lantuejoul, C. *Iterative Algorithms* in (1978), 85–89.

5. Khan, A. U. M., Torelli, A., Wolf, I. & Gretz, N. AutoCellSeg: Robust automatic colony forming unit (CFU)/cell analysis using adaptive image segmentation and easy-to-use post-editing techniques. *Scientific Reports* **8.** doi:10.1038/s41598-018-24916-9 (1 2018).

6. Carl, S. H., Duempelmann, L., Shimada, Y. & Bühler, M. A fully automated deep learning pipeline for high-throughput colony segmentation and classification. *Biology Open* **9.** doi:10.1242/bio.052936 (6 2020).

7. Geissmann, Q. OpenCFU, a New Free and Open-Source Software to Count Cell Colonies and Other Circular Objects. *PLOS ONE* **8,** 1–10. doi:10.1371/journal.pone.0054072. https://doi.org/10.1371/journal.pone.0054072 (Feb. 2013).

8. Bray, M. A., Vokes, M. S. & Carpenter, A. E. Using Cellprofiler for automatic identification and measurement of biological objects in images. *Current Protocols in Molecular Biology* **2015.** doi:10.1002/0471142727.mb1417s109 (2015).
